# Current Methods for Detecting Cell Membrane Transient Interactions

**DOI:** 10.3389/fchem.2020.603259

**Published:** 2020-12-07

**Authors:** Yousef Bagheri, Ahsan Ausaf Ali, Mingxu You

**Affiliations:** Department of Chemistry, University of Massachusetts, Amherst, MA, United States

**Keywords:** membrane biology, cell signaling, transient interactions, lipids, proteins, membrane probes

## Abstract

Short-lived cell membrane complexes play a key role in regulating cell signaling and communication. Many of these complexes are formed based on low-affinity and transient interactions among various lipids and proteins. New techniques have emerged to study these previously overlooked membrane transient interactions. Exciting functions of these transient interactions have been discovered in cellular events such as immune signaling, host–pathogen interactions, and diseases such as cancer. In this review, we have summarized current experimental methods that allow us to detect and analyze short-lived cell membrane protein–protein, lipid–protein, and lipid–lipid interactions. These methods can provide useful information about the strengths, kinetics, and/or spatial patterns of membrane transient interactions. However, each method also has its own limitations. We hope this review can be used as a guideline to help the audience to choose proper approaches for studying membrane transient interactions in different membrane trafficking and cell signaling events.

## Introduction

Cell membranes are composed of various lipid, protein, and carbohydrate compounds. These membrane components dynamically interact with each other to regulate cell cycles and communications (Lee, 2003; Contreras et al., 2011; Varshney et al., 2016; Sezgin et al., 2017). Based on the affinity and duration of these interactions, there are two types of membrane interactions: strong/stable and weak/transient. Strong/stable interactions last for a long time with binding affinities in the range of fM to nM. In contrast, weak/transient interactions happen only in the range of microseconds to seconds, with a μM–mM binding affinity (De Keersmaecker et al., 2018; Corradi et al., 2019). While it is worth mentioning that there is no absolute clear cut between these two types of membrane interactions, many membrane stable interactions have been well-studied, whereas in comparison, those transient interactions are often more challenging to investigate. This is because at any given time, only a small number of membrane transient interactions happen. The formed short-lived complexes are always under a dynamic equilibrium with monomers that will freely diffuse in the membrane. Meanwhile, these transient complexes are often disrupted or overlooked during *in vitro* isolation and purification processes.

In this review, we will first discuss the biological importance of these membrane transient interactions in regulating cellular functions. We will then focus on available experimental methods that can be used to study these transient interactions, especially in living cell membranes. We hope this review can provide some useful guidelines for future cell membrane studies.

## Importance of Membrane Transient Interactions

The first role of membrane transient interactions is to regulate the activation or suppression of many membrane protein complexes (Ikonen, 2008; Larsen et al., 2015; Sezgin et al., 2017). These protein complexes or oligomerizations cannot function without transient binding and interactions (Heldin et al., 1997; James et al., 2006). In membrane signaling network, the same protein is often involved in forming more than one complex and need to interact with different membrane partners. Weak and transient interactions will allow these membrane proteins to function in several signaling and trafficking pathways simultaneously. In addition, intracellular proteins can also be transiently recruited to the membrane to form dynamic receptor complexes (Sezgin et al., 2017; Corradi et al., 2019). Disruption of these fine-regulated membrane dynamic network will result in ineffective signal transduction and cell damage (Wymann and Schneiter, 2008).

**Figure 1:**
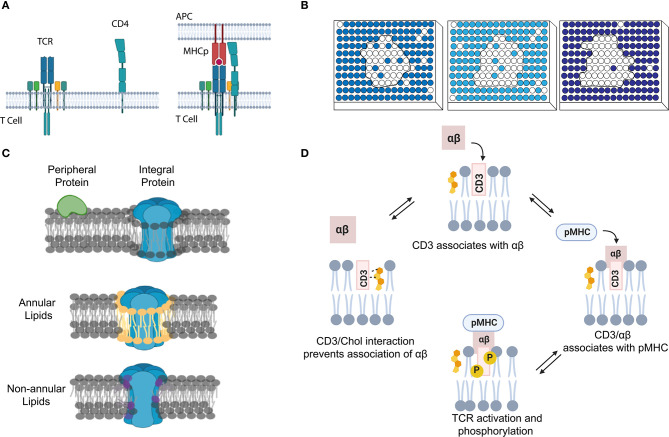
Transient interactions control membrane functions. **(A)** During T-cell signaling, the MHCp ligand binding induces dynamic protein–protein interactions between T-cell receptor (TCR) and CD4. **(B)** The strength of lipid–lipid interactions regulates membrane phase separation and domain formation. White circles represent that lipids prefer ordered domain. The darker the blue shading of circles, the poorer the ability of these disordered phase-preferred lipids to pack tightly. **(C)** The correlation between membrane protein location and their interactions with lipids. Membrane lipids can be thus categorized into three groups: bulk, annular, and non-annular. **(D)** Dynamic cholesterol interaction with TCR regulates its activation and prevents non-specific responses.

For example, the oligomerizations and functions of G-proteins and some electron transport complexes are controlled by membrane local environment (Nooren and Thornton, 2003; Acuner Ozbabacan et al., 2011; Sevcsik et al., 2015). G-proteins regulate various metabolic, developmental, humoral, and neuronal functions (Simon et al., 1991). Dependent on GTP and GDP levels, G-protein subunits (α, β, γ) can switch between stable binding and transient interaction to modulate cellular reactions (Neer and Clapham, 1988; Ritter and Hall, 2009). T-cell receptor (TCR) activation is also fundamentally regulated by membrane transient interactions of several protein subunits (Figure 1A). Immediately after T-cell engagement to the activating antigens, nanometer-sized TCR clusters are formed to function as a platform for the recruitment and activation of downstream effectors such as LAT and SLP-76 (Bunnell et al., 2002; Zal and Gascoigne, 2004; Yi et al., 2019).

In addition to these transient protein–protein interactions, lipid–protein and lipid–lipid interactions also play a major role in the cell membrane fluidity, curvature, and domain formation (Leikin et al., 1996; Aimon et al., 2014; McMahon and Boucrot, 2015). These short-lived and transient lipid-mediated interactions provide a mechanism allowing membrane compounds to quickly respond to stimuli, yet retain the ability to return to their original state. Based on the structure and degree of saturation, lipids tend to arrange themselves into either ordered or disordered domains (Bakht et al., 2007; Kaiser et al., 2009; Figure 1B). In the case that a membrane compound has similar binding affinities with several binding partners, by forming these lipid domains, the high chance of colocalization with a particular partner will clearly increase their opportunity of interactions. As a result, specific membrane complexes can form because of high local concentrations, despite their weak binding affinities.

For example, transient lipid interactions (in the range of μs–ms) have resulted in the formation of lipid rafts, i.e., small membrane domains that are usually <200 nm in diameter (Kolmakov et al., 2010; Smith, 2012; Sezgin et al., 2017; Bolmatov et al., 2020). Cholesterols, sphingolipids, and various proteins participate in these raft-like structures and regulate cellular processes such as in immune signaling, host–pathogen interaction, cancer, and cardiovascular diseases (Lorizate et al., 2013; Larsen et al., 2015; Varshney et al., 2016; Stone et al., 2017; Bolmatov et al., 2020).

Lipids can also directly interact with proteins to define their membrane structures and locations (Sezgin et al., 2017; Bolla et al., 2019). Dependent on the relative position of lipids to proteins, membrane lipids can be categorized into three groups: annular, non-annular, and bulk (Figure 1C; Lee, 2003; Contreras et al., 2011; Marius et al., 2012). Bulk lipids function as general membrane composition and usually have minimal interactions with membrane proteins (Lee, 2004). Annular lipids can form a ring-shaped shell that non-specifically surrounds membrane proteins (Contreras et al., 2011). In contrast, non-annular lipids can selectively occupy the cavities of proteins and function as specific building blocks or cofactors for the target proteins. Both annular and non-annular lipid–protein interactions are largely mediated by hydrophobic interactions. Meanwhile, hydrophilic interactions between polar amino acid chains and lipids' head groups have further increased the affinity and specificity of these lipid–protein conjugates (Lee, 2004; Contreras et al., 2011). Compared to the transient annular lipid–protein interactions, many non-annular lipid–protein interactions are more stable and long-lasting (Lee, 2011; Marius et al., 2012), e.g., as that happens during the selective interactions between phosphatidylglycerol and the homotetrameric potassium channel KcsA (Heginbotham et al., 1998; Valiyaveetil et al., 2002).

We can also distinguish membrane lipid–protein interactions based on the different categories of membrane proteins (Figure 1C). Integral membrane proteins and lipid-anchored proteins can interact with lipids through hydrophobic interactions. While peripheral proteins will normally interact with lipids through transient binding with lipids' head groups, different strengths of these interactions can be used to define proteins' locations and functions. For example, proteins that specifically bind glycosphingolipids or sphingomyelin can be recruited to the aforementioned membrane nanodomains (Fantini and Yahi, 2010; Pontier and Schweisguth, 2012; Sezgin et al., 2017). During T-cell activation, cholesterol can selectively bind to the resting TCR and activate the allosteric transition of the TCR complex (Swamy et al., 2016; Figure 1D). Meanwhile, the dynamic interactions between CD28 and cholesterol or sphingomyelin also help recruit these membrane proteins into the same submembrane domains of the TCR complex for the efficient T-cell activation (Kabouridis et al., 2000; Yang et al., 2017). These transient lipid–protein interactions are required to prevent spurious TCR activation and ensure accurate membrane functions.

Membrane lipid domains are also involved in cancer development and progression. It has been shown that oncogenic proteins such as mucin 1 and urokinase plasminogen activator surface receptor are centered in raft-like domains (Staubach et al., 2009). Mitogenic signaling is also initiated from cell surface receptors in the lipid domains (Heldin et al., 2016; Varshney et al., 2016). In addition, by disrupting raft-like membrane domains, anticancer drugs can be developed (Gajate and Mollinedo, 2007). Similarly, lipid domain partitions have also been observed in other health threats such as vascular diseases (Maguy et al., 2006). Membrane transient interactions are indeed important cellular events that require better understanding.

## Methods to Study Membrane Transient Interactions

In the past few decades, several computational and experimental methods have been developed to study membrane transient interactions (Loura et al., 2010; Smith, 2012; Corradi et al., 2019). With significant improvement in the computing power and theoretical models, computational approaches can now predict and explain many membrane short-lived interactions. Dynamic simulations have been applied to study various membrane interactions (Corradi et al., 2019; Muller et al., 2019), such as the effect of lipid environment on membrane channel functions (Gu and de Groot, 2020), the impact of polyunsaturated fatty acids on lipid raft structure and distribution (Levental et al., 2016), and lipid-induced cross-talk through the leaflets (Bossa et al., 2015). There are several great reviews on using computational methods to study membrane dynamic interactions (Corradi et al., 2019; Muller et al., 2019; Siebenmorgen and Zacharias, 2020). Here, we will mainly discuss experimental methods that can be used to really detect and analyze transient interactions in the cell membranes.

### Nuclear Magnetic Resonance Spectroscopy

X-ray crystallography and electron microscopy can provide valuable structural information about stable membrane protein–lipid complexes (Zhou et al., 2001; Nooren and Thornton, 2003). However, these methods are normally not suitable for studying transient interactions or conformational changes. In comparison, nuclear magnetic resonance (NMR) spectroscopy is better for analyzing structure and dynamics of membrane transient complexes (Díaz-Moreno et al., 2005; Dannatt et al., 2016; Purslow et al., 2020). Thermodynamic information such as the binding affinities of membrane partners can also be measured in NMR (Purslow et al., 2020). NMR is a powerful technique for studying membrane transient interactions, especially if atomic-level structural information is needed.

**Figure 2:**
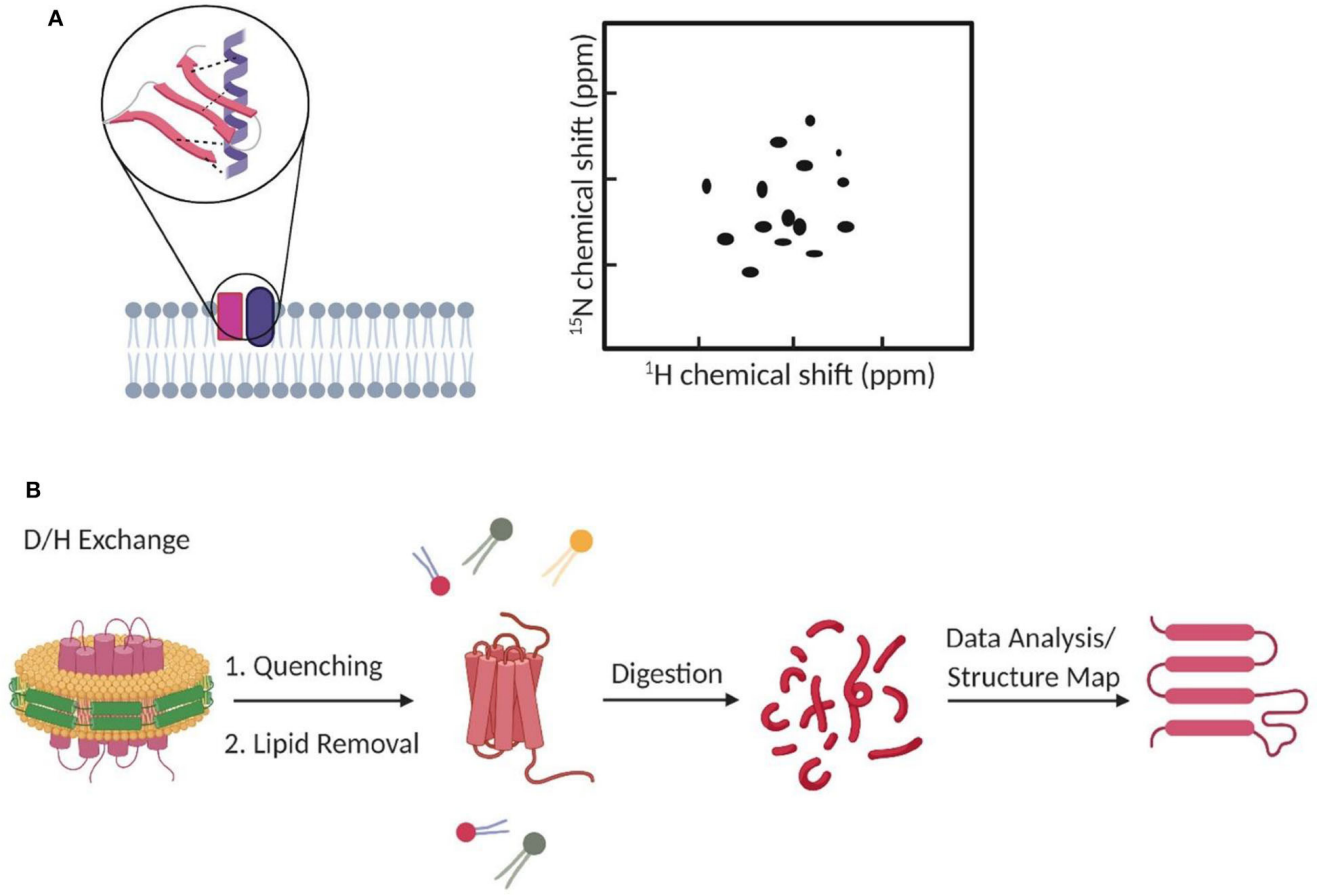
The use of NMR and mass spectrometry for studying membrane transient interactions. **(A)** Dynamic membrane protein structural changes and interactions can be analyzed from the observed chemical shifts in 2D NMR spectrum. **(B)** Schematic of a general HDX-MS workflow to study protein conformation changes induced by dynamic lipid–protein interactions.

Chemical shift of proteins is very sensitive to the local electronic environment. As a result, changes in the chemical shift can be used for the NMR analysis of protein complex formation and structure (Ahuja et al., 2013; Figure 2A). For example, chemical shift perturbation is a suitable method for studying transient interactions with μM–mM binding affinities (Acuner Ozbabacan et al., 2011). In this method, a ^15^N- or ^13^C-labeled target protein is titrated with different amounts of unlabeled binding partner. Changes in the two-dimensional (2D) heteronuclear single-quantum coherence spectra are then used to calculate the binding affinity and binding site of the protein complex. Several other NMR-based methods have also been developed to study membrane dynamic protein–protein interactions, such as solvent paramagnetic relaxation enhancement, residual dipolar coupling, and nuclear Overhauser effect (Vinogradova and Qin, 2012; Gell et al., 2017; Larsen et al., 2018).

However, it is still difficult to use NMR to characterize structures of large-molecular-weight complexes. Appropriate kinetic models should also be carefully chosen to convert the obtained NMR data into accurate interaction parameters (Furukawa et al., 2016). In this regard, a relevant strategy based on site-directed spin labeling electron paramagnetic resonance (EPR) spectroscopy can be advantageous for the more direct study of membrane transient interactions (Subczynski et al., 2010; Zhang et al., 2011). However, both NMR and EPR are still challenging to be directly applied for studying interactions in live cell membranes. The differences between real cell membrane environment and *in vitro* conditions have to be considered.

### Mass Spectrometry

Mass spectrometry (MS) is another common method to study membrane dynamic interactions (Konijnenberg et al., 2014; Gupta et al., 2017, 2018; Bolla et al., 2019; Frick and Schmidt, 2019). MS does not provide high-resolution target structure, but it can measure the mass and stoichiometry of the complex (Pyle et al., 2018; Frick and Schmidt, 2019). Among different types of MS, hydrogen–deuterium exchange MS (HDX-MS) has gained much attention for studying transient (millisecond range) protein conformational changes (Rist et al., 2005; Giladi and Khananshvili, 2020). For example, HDX-MS has been used to study how transient lipid–protein interactions can induce conformational and functional changes of bacterial leucine transporter LeuT (Adhikary et al., 2017), rhomboid protease GlpG (Reading et al., 2017), and various secondary transporters (Martens et al., 2018). In HDX-MS, labile hydrogens in the protein complex can be exchanged with deuterium. The mass uptake at different locations of the complex will then be measured and mapped to allow visualization of its structural dynamics (Figure 2B; Martens et al., 2019; Giladi and Khananshvili, 2020). High-purity samples are desirable but not essential, because the contaminants can be easily ruled out during data processing (Martens et al., 2019).

HDX-MS is still limited with some shortcomings. First, HDX-MS reports the changes in the protein uptake of deuterium, not directly the complex conformation. For a 50-kDa protein, analyzing and converting one set of data to the conformational changes may take ~2 days (Martens et al., 2019). Detergents are normally used to extract membrane protein complexes in MS studies. However, affected by the choice of detergent, the obtained results may not represent the native structure of membrane complexes. Membrane-mimic lipid nanostructures, such as picodiscs, nanodiscs, styrene maleic acid lipid particles, bicelles, and liposomes (Grinkova et al., 2010; Dürr et al., 2012; Marty et al., 2016; Frick and Schmidt, 2019), have been developed to alleviate this problem, while the effect of these artificial lipid structures should be still carefully considered.

Secondary ion MS (SIMS) has been used to image lipid distributions in the cell membranes. For example, by metabolically incorporating isotopic form of lipids, SIMS allows the imaging of sphingolipid and cholesterol distributions in the membrane domains, with a resolution of <100 nm (McQuaw et al., 2007; He et al., 2017; Kraft, 2017). Compared to fluorescent labeling as mentioned below, such isotope labeling has less impact on the natural membrane properties of the target lipids. SIMS can indirectly verify lipid–lipid or lipid–protein interactions by quantifying target lipid distributions and transitions upon addition and depletion of the partner lipids or proteins (McQuaw et al., 2007; He et al., 2017). However, SIMS cannot directly assess membrane interactions, especially those being transient. In addition, SIMS is not suitable for live-cell studies. Cells need to be dehydrated first, and the measurements are performed in vacuum.

### Fluorescence Correlation Spectroscopy and Cross-Correlation Spectroscopy

The development of fluorescence techniques has revolutionized membrane biophysical studies (Triffo et al., 2012; Martinez-Moro et al., 2019). Fluorescence methods enable the real-time study of dynamic phenomena in living cell membranes with high temporal and spatial resolution. Here, we will discuss some fluorescence spectroscopy and microscopy approaches that have been used to study transient interactions in the cell membranes.

The first method is based on fluorescence correlation spectroscopy (FCS). FCS can be used to analyze the concentrations, motilities, and interactions of membrane fluorescent-labeled compounds (Haustein and Schwille, 2003; Bacia et al., 2006). It works by measuring fluorescence fluctuations in a tiny focal spot. Membrane complex formation results in changes in the mobility. In order to obtain reliable interaction information in FCS, a large-molecular-weight difference (usually >eight-fold) between bound and unbound state is needed. To study membrane interactions between molecules of similar molecular weight, fluorescence cross-correlation spectroscopy (FCCS) can be a good option. FCCS measures the fluctuations and correlations of two fluorescent-labeled partners in the same focal spot (Bacia et al., 2006; Bacia and Schwille, 2007). If these two molecules interact, even transiently, they will diffuse together and exhibit a positive cross-correlation signal.

**Figure 3:**
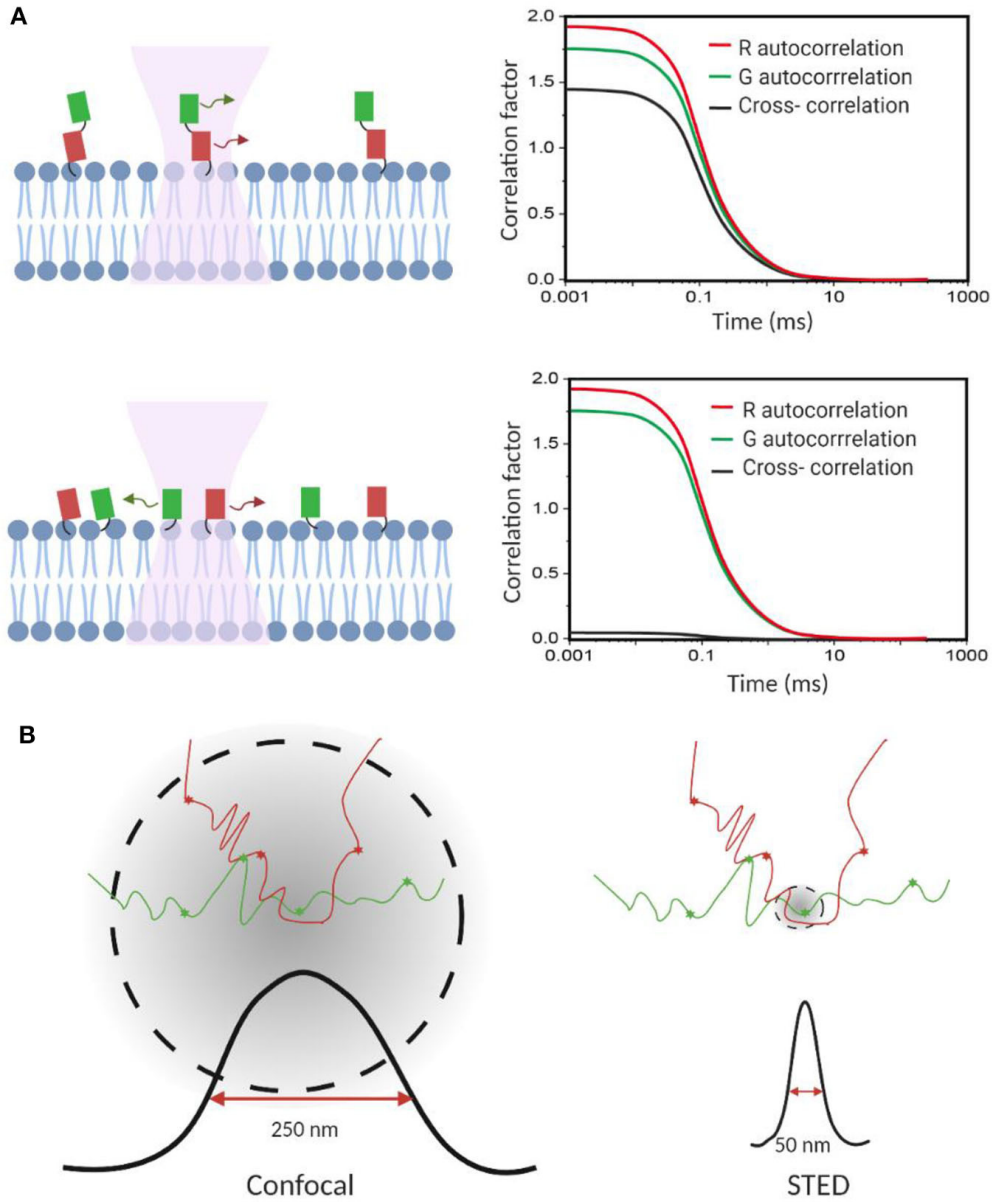
Fluorescence correlation spectroscopy measures membrane transient interactions at the single-molecule level. **(A)** Schematic of fluorescence cross-correlation of correlated and uncorrelated protein diffusions in the membrane. Increase in correlation among targets results in higher cross-correlation. **(B)** STED-FCS provides better spatial resolution to distinguish target interactions than the conventional confocal FCS.

FCCS has become a popular method to study, at the single-molecule level, membrane dynamic interactions (Bacia et al., 2006; Sadamoto and Muto, 2013; Ma et al., 2014; Martinez-Moro et al., 2019). FCCS is able to report the correlated diffusion and binding stoichiometry over multiple time scales (10^−7^-10^1^ s) (Bacia et al., 2006). For example, FCCS has been used to study the regulation effect of cardiolipin and phosphatidylglycerol on the dynamic oligomerization of mitochondrial membrane voltage-dependent anion channel (Betaneli et al., 2012). In another example, SNARE proteins were found to prefer incorporating into disordered membrane domains (Bacia et al., 2004). As an example for measuring transient protein–protein interactions in live cell membranes, pulsed-interleaved excitation FCCS was used to investigate anchor-mediated dynamic colocalization of lymphocyte cell kinase, RhoA, and K-Ras proteins (Figure 3A; Triffo et al., 2012).

FCS and FCCS are not suitable for monitoring slowly moving membrane particles, though. In this case, the number of interactions within the focal spot is hardly sufficient to obtain good statistics; meanwhile, the fluorophores can be easily bleached before leaving the focal spot (Triffo et al., 2012). Traditional FCCS can assess only tiny focal spots rather than the whole membrane area. To assess a large membrane, temporal and spatial correlation analysis can be combined with scanning FCS/FCCS now (Ruan et al., 2004).

### Super-Resolution Microscopy

FCS and FCCS normally operate on a confocal microscope. However, limited by the relatively big focal spot (~200 nm in diameter), it is challenging to validate if these membrane interactions indeed happen on the molecular level, especially in those small nanodomains. Stimulated emission depletion (STED) microscopy is a super-resolution approach that can be combined with FCS to image interactions below the diffraction limit (Honigmann et al., 2014; Saka et al., 2014; Sevcsik et al., 2015; Sezgin et al., 2019). Using STED-FCS, a 20-nm focal spot is accessible, which allows a better distinction between membrane free diffusion and transient interactions (Eggeling et al., 2009).

STED-FCS has been used to monitor cholesterol-dependent membrane diffusion and colocalization of sphingolipids and glycosylphosphatidylinositol (GPI)–anchored proteins in living cells (Eggeling et al., 2009; Figure 3B). These lipid moieties were found to be transiently trapped in cholesterol-mediated complexes in a <20-nm-diameter area. In a more recent study, the same authors applied scanning STED-FCS to investigate the spatial distributions of these membrane cholesterol–lipid interactions. Again, transient interaction hotspots across the cell membrane were observed (Honigmann et al., 2014). Dynamic cholesterol-dependent multiprotein membrane assemblies can also be visualized with STED-FCS (Saka et al., 2014).

In addition to STED, other single-molecule imaging and super-resolution techniques, such as total internal reflection fluorescence (TIRF) microscopy, structured illumination microscopy, and photoactivated localization microscopy, have also been used to study membrane transient interactions (Hess et al., 2007; Galbraith and Galbraith, 2011). Considering that the short-lived interactions usually do not result in many accumulated signals over a large membrane plane, microscopes of high spatial resolution and single-molecule sensitivity are useful techniques in our understanding of these heterogeneous and transient interactions. However, μs–ms range membrane transient interactions are often still too fast to be analyzed based on single-molecule imaging or colocalization methods. Many super-resolution techniques have to sacrifice the temporal resolution to obtain spatially resolved information (Westphal et al., 2008; Galbraith and Galbraith, 2011).

**Figure 4:**
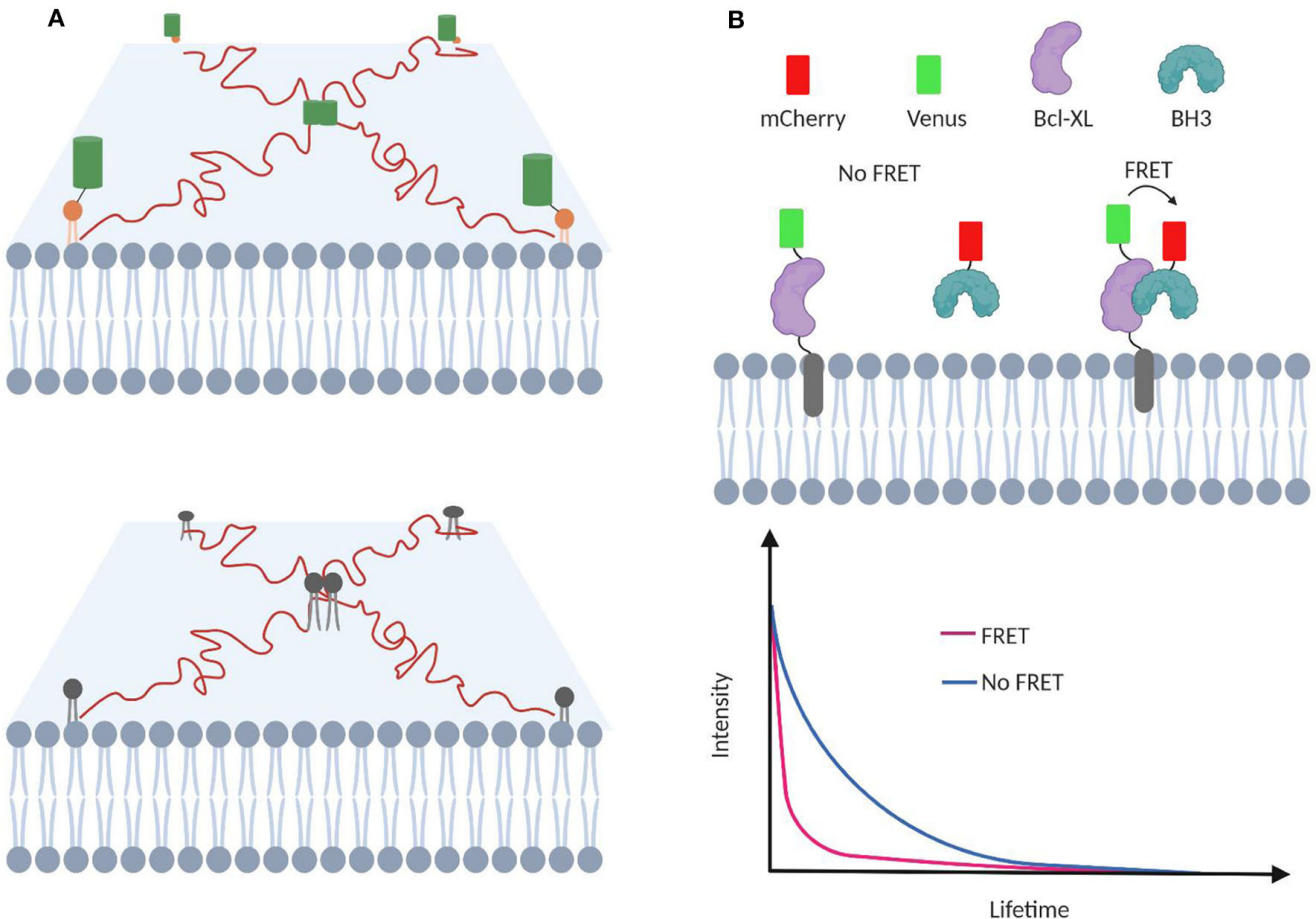
TIRF-based single-molecule tracking to investigate membrane transient interactions. **(A)** Protein homodimerization and lipid–lipid interactions are transient process that can be studied with TIRF. **(B)** Transient interactions between proapoptotic BH3 protein and membrane-bound antiapoptotic Bcl-2 proteins can be studied using FLIM-FRET. FRET among Venus and mCherry facilitates Venus relaxation and lowers its lifetime.

With fast image acquisition, high signal-to-noise ratio, and spatial resolution, TIRF is a popular approach to monitor dynamic motions and interactions in live cell (Suzuki et al., 2012; Stender et al., 2013). Using bright and photostable fluorophores, transient colocalization as short as microseconds can be detected in TIRF. For example, transient homodimerization of GPI-anchored proteins was found as critical organization unit for membrane domain formation (Suzuki et al., 2012; Figure 4A). While, interestingly, somewhat controversial result was shown in another TIRF-based study, which indicated that GPI-anchored proteins were not found to partition in the membrane domains (Sevcsik et al., 2015), this discrepancy may indicate that it is still challenging to precisely measure membrane transient interactions based on simply monitoring the trajectory of each single molecule.

Meanwhile, because normally TIRF is performed close to the surface of the coverslip, it is mainly capable of studying the surface-attached part of the target cells (Hell et al., 2015), e.g., the basal surface of epithelial cells but not the apical side. Considering that basal and apical cell membrane may have different compositions and functions, TIRF may only provide partially representative information about the whole cell membrane. Furthermore, close proximity with the glass surface may also induce non-natural protein/lipid organization and diffusion patterns.

### Förster Resonance Energy Transfer

Förster resonance energy transfer (FRET) is another powerful technique to investigate membrane molecular interactions (Rao and Mayor, 2005; Loura et al., 2010; Loura and Prieto, 2011). By modifying the target membrane partner with a donor and acceptor fluorophore, respectively, once two molecules are in close proximity (<10 nm), the energy transfer between the donor and acceptor results in a strong FRET signal. For example, FRET has been used to discover specific membrane interactions between sphingomyelin and the p24 protein (Contreras et al., 2012), as well as the membrane domain formation during the interactions of N-Ras and K-Ras4B oncoproteins (Li et al., 2019).

FRET is often combined with fluorescence lifetime imaging microscopy (FLIM) to study membrane transient interactions (Llères et al., 2007; Liu et al., 2012; Gagnon et al., 2017). In FLIM-FRET, the energy transfer between the FRET donor and acceptor results in a fluorescence lifetime decrease of the donor fluorophore. Compared with fluorescence intensity-based measurement, FLIM-FRET is advantageous of being independent of the membrane concentration of target molecules (Wallrabe and Periasamy, 2005). This feature is critical considering the heterogeneity and diverse distribution patterns of lipids and proteins. For example, the effect of various mutations and chemical inhibitors in modulating transient interactions between membrane-bound antiapoptotic Bcl-2 protein and proapoptotic BH3 protein has been measured using FLIM-FRET (Liu et al., 2012; Osterlund et al., 2015; Figure 4B).

In addition to FRET, distance-dependent non-FRET quenching has also been used to track transient interactions in live cell membranes (Artetxe et al., 2013). For example, fluorophore- and quencher-labeled lipid pairs were used to study the impact of head group size of sphingolipids and ceramide on their interactions with membrane cholesterols (Artetxe et al., 2013). In these FRET or quencher approaches, both target molecules are needed to be fused with fluorophore or quencher at a specific location to ensure close proximity during interactions. Meanwhile, the limited signal-to-noise ratio has prevented the application of these FRET methods for imaging some really transient (submillisecond) membrane interactions.

### Membrane Two-Hybrid Assays

**Figure 5:**
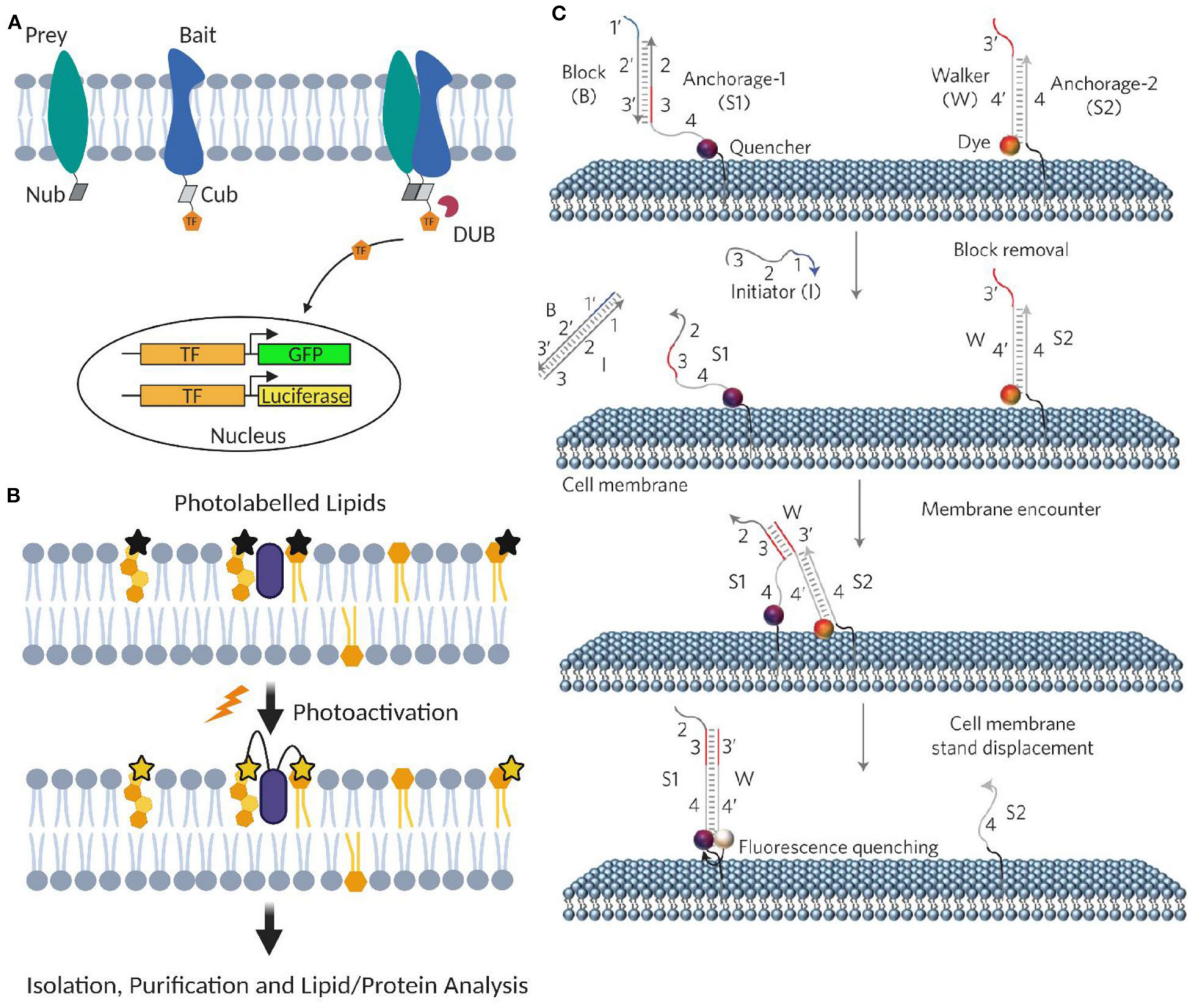
Artificial membrane probes for studying dynamic membrane interactions. **(A)** Schematic of membrane two-hybrid assays for characterizing membrane protein–protein interactions based on the formation of deubiquitinating enzyme (DUB). **(B)** Schematic of applying photoactivatable lipid probes for proteome-wide mapping of membrane lipid–protein interactions. **(C)** Schematic of the toehold-mediated DNA displacement reaction for monitoring membrane lipid–lipid interactions. Reprinted with permission from You et al. (2017). Copyright (2017) Springer Nature Limited.

Yeast and mammalian two-hybrid assays have been developed to characterize membrane protein–protein interactions, including transient ones (Snider et al., 2010; Petschnigg et al., 2014). In these assays, each half of a split ubiquitin is tagged, respectively, to the target membrane protein (named as bait and prey). Once bait and prey proteins interact, a pseudoubiquitin is formed and then cleaved by cytosolic deubiquitinating enzymes to release a reporter protein, such as a transcription factor for generating fluorescent proteins or luciferases (Petschnigg et al., 2014; Figure 5A).

One major advantage of membrane two-hybrid assay is that it can be used for high-throughput screening. For example, using this approach, CRKII has been identified as a new adaptor protein that can dynamically interact with membrane epidermal growth factor receptor in oncogenic signaling (Petschnigg et al., 2014). These membrane two-hybrid assays can also potentially be useful in discovering drug molecules that can interfere with transient protein interactions during membrane signaling.

On the other hand, compared with the reversible FRET interactions, these membrane two-hybrid assays are normally irreversible and not suitable to image the location or dynamics of membrane interactions. The relatively high rate of false-positive signals, e.g., from protein overexpression, requires careful data analysis and validation. In theory, other methods that can convert dynamic interactions into permanent signals, such as *in situ* proximity ligation assay (Petschnigg et al., 2014) and bimolecular fluorescence complementation (Kodama and Hu, 2012), may also be useful for characterizing membrane transient interactions. However, the interaction time required for the proximity-based ligation or reconstitution of fluorescent proteins made these methods practically challenging to study short-lived membrane interaction events.

### Photoactivatable Lipid Probes

A powerful method to profile proteome-wide membrane lipid–protein interactions is based on photoactivatable lipid probes (Xia and Peng, 2013; Peng et al., 2014). In this method, after incorporating into the membranes and upon light irradiation, photoactivatable lipids can be activated and cross-linked with their interacting membrane protein partners (Figure 5B). Transiently formed membrane lipid–protein complexes can thus be covalently stabilized. After separation and purification, different approaches such as MS, autoradiography, high-performance liquid chromatography, and gel electrophoresis can be used to analyze the cross-linked membrane complexes.

These photoactivatable lipid probes can be used in both artificial and live cell membranes. They have been used to proteome-wide map and identify new membrane proteins that can transiently bind target lipids (Niphakis et al., 2015). Without prior knowledge, unlabeled endogenous membrane proteins can be directly cross-linked with the photoactivatable lipid probes once an interaction happens. Meanwhile, with the help of bifunctional lipid probes, the binding sites of these lipid–protein complexes can also be determined (Xia and Peng, 2013; Peng et al., 2014). In addition to membrane lipid–protein interactions, these photoactivatable probes can also be used to map dynamic lipid-anchored protein–protein interactions.

On the other hand, by introducing several functional groups in the lipid molecules, the natural interaction pattern of the target lipid may be disrupted. In addition, the crosslinking efficiency is highly dependent on the activity and position of the functional group. As a result, the choice of bio-orthogonal crosslinking moiety and tagging site in lipids is critical in developing accurate and efficient photoactivatable probes. A series of photoactivatable lipid probes have been developed (Xia and Peng, 2013). With continuous efforts in the characterization and optimization of these functional probes, the photo-crosslinking approach can play a critical role in profiling membrane transient interactions.

### DNA-Based Membrane Probes

We have recently developed a DNA-based approach to image transient interactions in live cell membranes (You et al., 2017). In this approach, individual membrane lipid–lipid and protein–protein interactions are converted into changes in the collective fluorescence signal, which can be easily detected by fluorescence microscopes. DNA probes can be covalently modified onto target membrane lipids without affecting their lateral diffusion and domain partition (You et al., 2017; Bagheri et al., 2019a). The highly precise and controllable DNA hybridizations have further allowed programmable manipulation and function of these membrane-anchored DNA probes (Bagheri et al., 2019b; Zhao et al., 2020).

Our DNA probe is realized through a toehold-mediated strand displacement reaction (Figure 5C). To measure membrane transient lipid–lipid interactions, two target lipids were modified with a quencher strand (S_1_) and an unmodified anchor strand (S_2_), respectively. Once two lipids are located within ~5 nm, a fluorophore-labeled DNA probe (W) will translocate from S2 to S1, resulting in a fluorescence quenching. By monitoring membrane fluorescence signal changes, this approach allows live-cell imaging of μs-to-ms-range lipid–lipid interactions using common fluorescence microscope and flow cytometer. Similar strategy can also be applied to monitor membrane protein–protein interactions using target protein-specific DNA aptamers (You et al., 2017).

This modular DNA-based approach can be easily adopted to different targets on the cell membrane. However, it is necessary to carefully evaluate the effect of DNA modification on the membrane properties and behaviors of the target molecules. Furthermore, the membrane densities of the DNA probes should be kept in the minimum possible level to provide enough fluorescence signals but also avoid membrane interruptions.

## Discussion

Membrane transient interactions are important biophysical events for membrane trafficking, signal transduction, and cell proliferation (Ikonen, 2008; Kraft, 2013; Sezgin et al., 2017; Martens et al., 2018; Yi et al., 2019). With fast emergence of new powerful methods and tremendous advancement in instrumentations, our understanding of these short-lived events has been significantly improved. As shown in Table 1, we have summarized and compared different properties and applications of these variant experimental methods in studying membrane transient interactions. We will discuss as follows our view on how to further advance these methods, as well as future directions in membrane interaction studies.

**Table 1:** Currently available methods for studying cell membrane transient interactions.

**Method**	**Resolution**		**Membranes applied**		**Interactions studied**	**Interaction information provided**
	**Temporal**	**Spatial**	**Cell**	**Synthetic**		
NMR	ms	Atomic	No	Yes	Lipid–protein Protein–protein	Structure and conformation Kinetic and equilibrium constant
HDX-MS	ms	μm	No	Yes	Lipid–protein Protein–protein	Protein conformation Stoichiometry
SIMS	min	100 nm	Yes	Yes	Lipid–lipid Lipid–protein	Membrane distribution
FCS and FCCS	μs	200 nm	Yes	Yes	Lipid–lipid Lipid–protein Protein–protein	Membrane concentration Membrane distribution Kinetic and equilibrium constant
STED-FCS	μs	20 nm	Yes	Yes	Lipid–lipid Lipid–protein Protein–protein	Membrane concentration Membrane distribution Kinetic and equilibrium constant
TIRF	μs	20 nm	Yes	Yes	Lipid–lipid Lipid–protein Protein–protein	Membrane distribution Kinetic and equilibrium constant
FRET	μs	10 nm	Yes	Yes	Lipid–lipid Lipid–protein Protein–protein	Membrane distribution Kinetic and equilibrium constant
FLIM-FRET	s	10 nm	Yes	Yes	Protein–protein	Membrane distribution Kinetic and equilibrium constant
MTH	s	200 nm	Yes	Yes	Protein–protein	High-throughput screening
PAL	ms	μm	Yes	Yes	Lipid–protein	Proteome-wide profiling Protein–lipid complex structure
DNA probe	μs	10 nm	Yes	Yes	Lipid–lipid Protein–protein	Membrane distribution Kinetic and equilibrium constant

*MTH, membrane two-hybrid assay; PAL, photoactivable lipid probe*.

NMR and MS are both almost label-free approaches that can measure membrane interactions in their natural forms. However, direct *in situ* analysis in live cell membranes is still difficult for both methods. The recent development of SIMS seems to provide a promising solution for this issue. Furthermore, it is challenging to apply NMR and MS to study really fast (< ms) membrane interactions or conformational changes. Indeed, efforts have been taken to improve the temporal resolution of MS, for example, by conjugating with a flow quenching system (Rist et al., 2005; Giladi and Khananshvili, 2020).

NMR provides great spatial resolution for structural studies. In comparison, MS and other methods usually suffer from low spatial resolutions. To potentially improve their resolution, complementing the experimental data with computational simulations can be an effective approach to obtain molecular-level structural information (Martens et al., 2019).

Fluorescence-based methods allow direct and real-time visualization of transient interactions in live cell membranes. Using commercially available microscopes, currently it is easy to precisely measure ms–s range membrane interactions. Meanwhile, advanced imaging systems have been designed and applied for detecting even faster (< μs) interactions (Simmons and Konermann, 2002; Rist et al., 2005; Honigmann et al., 2014; Sezgin et al., 2019). We are expecting these advanced systems to be more readily available to the broad community of researchers in the future.

With the development of versatile fluorescent membrane probes, advanced fluorescence microscopes have genuinely reshaped our perception of membrane transient interactions. However, methods that allow high-throughput and quantitative analysis are still limited, especially for screening compounds that can potentially regulate critical transient interactions in diseases. As a result, membrane heterogeneities and cell-to-cell variations of these dynamic interactions or so-called membrane “interactome” are largely unknown.

Even using the same method, contradictory results have been obtained for many transient interactions such as in the case of membrane nanodomains. This inconsistency may result from the limited precision of the methods, but also is likely due to the dynamic nature of these transient interactions. Under different cellular environment and conditions, the lifetime of membrane interactions can be varied dramatically. Standard experimental procedure and statistical data analysis should be established. Meanwhile, it will be helpful to use more than one method to validate the observed results for membrane transient interactions.

A systematic comparison of the accuracy and performance of available methods in analyzing membrane transient interactions is highly desired. Most laboratories chose the methods based on their own experience and expertise. One goal of this review is to provide the first step in initiating such comparison. We believe once a well-accepted membrane model and standard approach were established, versatile functions of transient interactions in regulating membrane trafficking and cell signaling can be discovered in both healthy and diseased cell conditions.

This review has been focusing on major methods in studying transient membrane interactions among individual lipids and proteins. Other interesting membrane interactions, such as membrane vesicle formation, transportation, and fusion, are also critical for various cellular functions such as cell growth, hormone secretion, and neurotransmission (Novick and Schekman, 1979; Wickner and Schekman, 2008; Rupert et al., 2017; Welsh et al., 2017). Unfortunately, because of the limited space in this article, we are not able to cover all these topics. We would like to refer interested audience to those reports mentioned above.

In short, the introduction of new methodologies has been continuously improving our knowledge of transient membrane interactions. It is important that researchers with different expertise to collaboratively work together in developing more powerful toolkits. We believe that future evolution of methods and instrumentations will continue to play a major role in advancing our understanding of the dynamic nature of cells and other biological system.

## Author Contributions

MY provided the guidance for the whole project and revised the manuscript. YB and AA searched the literature and wrote the manuscript. All the authors have reviewed, edited, and approved the manuscript before submission.

## Conflict of Interest

The authors declare that the research was conducted in the absence of any commercial or financial relationships that could be construed as a potential conflict of interest.
